# Water Scarcity and Water Quality: Identifying Potential Unintended Harms and Mitigation Strategies in the Implementation of the Biosand Filter in Rural Tanzania

**DOI:** 10.1177/1049732320918860

**Published:** 2020-05-25

**Authors:** Lise Hovden, Tina Paasche, Elias Charles Nyanza, Sheri Bastien

**Affiliations:** 1 Oslo University Hospital, Oslo, Norway; 2 Bø municipality, Vesteralen, Norway; 3 Catholic University of Health and Allied Sciences, Mwanza, Tanzania; 4 Norwegian University of Life Sciences, Ås, Norway; 5 University of Calgary, Calgary, Alberta, Canada

**Keywords:** water quality, water scarcity, Biosand Filter, unintended harms, Tanzania, qualitative methods

## Abstract

Bottom-up public health interventions are needed which are built on an understanding of community perspectives. Project SHINE is a community-based participatory action research intervention focused on developing sustainable water, sanitation, and hygiene strategies with Maasai pastoralists in Tanzania. The aim of the study is to understand perceptions related to water quality and scarcity as well as to assess the potential of the Biosand Filter as a low-cost, low-tech water treatment option. To avoid unintended harms, the community was engaged in identifying potential harms and mitigation strategies prior to the implementation of the filter.Two in-depth interviews and two group discussions were analyzed using thematic content analysis, while three think tanks were analyzed using directed content analysis. The findings highlight a range of concerns regarding water scarcity and quality. The think tank approach was an effective means of engaging the community in identifying potential unintended harms across four dimensions: the physical, psychosocial, economic, and cultural contexts. In addition, two external themes emerged as salient: political harm and harm by omission.

## Background

Globally, approximately 1.8 billion people rely on a source of drinking water that is fecally contaminated (World Health Organization [WHO], 2015). Ensuring access to safe water is one of the most effective measures to promote health and reduce poverty in low- and middle-income countries (LMIC) (WHO, 2015). Access to safe water is prominent on the international agenda as evidenced most recently in the Sustainable Development Goals (SDGs) in which the aim of Goal 6 is to “ensure availability and sustainable management of water and sanitation for all” (United Nations, 2016). To achieve this requires investments in infrastructure, sanitation facilities, and improved hygiene practices.

At the household level, ensuring access to clean water is often promoted through the use of treatment technologies, such as the Biosand Filter (BSF) in LMICs (United Nations Development Program, 2016). The BSF is one of the most effective household water treatment options as it is effective in decreasing waterborne disease and death (Sobsey et al., 2008). A review of four trials examining the health impact of the concrete BSF found that the BSF can reduce diarrheal disease by 50% or more (Stauber et al., 2014).

However, introducing any new technology, regardless of its promise, in a resource-constrained community may lead to potential unintended harms, as is possible even with carefully planned and well-intentioned public health interventions (Allen-Scott et al., 2014; McQueen, 2014). Unintended harms are rarely reported in the literature (Allen-Scott et al., 2014; Lorenc & Oliver, 2014) and, thus far, there has been a lack of frameworks with which to address this challenge associated with interventions. For an intervention to be successful, there is a need to take contextual factors into account when planning for implementation, for instance, the unique social determinants affecting health within a community as well as the needs, assets, and capacities of the target population (Davies & Macdowall, 2006; Glanz & Bishop, 2010).

**Figure 1.**
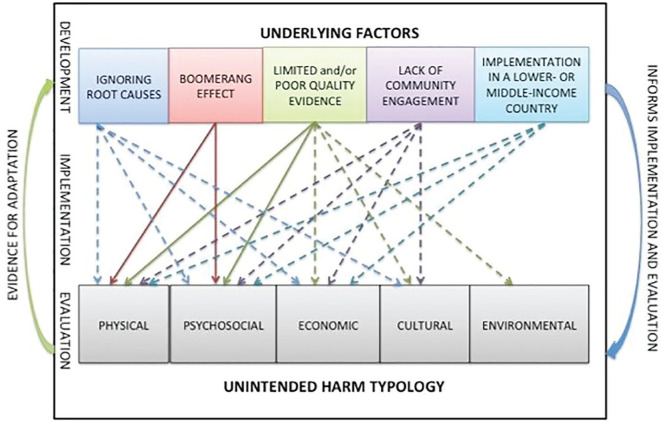
A concept map illustrating the relationships between the unintended harm typology and emergent underlying factors. *Note.* Solid lines indicate higher levels of evidence to support the underlying factors and typology relationship. Dashed lines indicate the presence, yet limited evidence on the underlying factor and typology relationship (Allen-Scott et al., 2014, p. 11).

An unintended harm typology developed by Allen-Scott and colleagues (2014) was applied as an overarching framework in this study to develop an understanding of potential unintended harms associated with the BSF implementation. As depicted in Figure 1, this typology was developed through a scoping review of studies that reported unintended harms related to public health interventions with five categories of unintended harms: (a) physical, (b) psychosocial, (c) economic, (d) cultural, and (e) environmental. These are in addition to the following underlying factors: (a) *ignoring root causes*, (b) *prevention of one extreme leads to another extreme (boomerang effect)*, (c) *limited and/or poor-quality evidence*, (d) *lack of community engagement*, and (e) *implementation in an LMIC* (Allen-Scott et al., 2014).

Project SHINE (Sanitation and Hygiene INnovation in Education) is a school and community-based participatory action research intervention to develop culturally relevant and effective water, sanitation, and hygiene strategies among Maasai pastoralists in rural Tanzania (Bastien et al., 2015; Hetherington et al., 2017). Formative research conducted prior to the implementation of the intervention indicated that water scarcity and water quality were substantial concerns in the community. A cross-sectional survey completed as part of Project SHINE among 175 households in the Ngorongoro Conservation Area (NCA), in which the study took place, indicated that while a substantial proportion of respondents reported they have access to an improved water supply, access to improved sanitation facilities was lower than the national average for rural areas, and open defecation is still commonly practiced (Nyanza et al., 2018). In addition, local hospital records indicated that soil-transmitted helminth infections and protozoa are among the top 10 diagnoses in the region (Bastien et al., 2015). It is against this backdrop that a pilot study of the BSF as a low-cost, low-tech water treatment option was conducted. The BSF was selected based on a robust evidence base for use in water that is highly turbid, such as is the case in the NCA. The data reported here all stem from the preimplementation phase.

The aims of the present study are, first, to explore community perceptions related to water scarcity and quality and, second, to engage the community in identifying potential unintended harms and mitigation strategies related to the implementation of the BSF. In-depth interviews and group discussions were used to address the first aim using a more flexible, open-ended approach, whereas think tanks were used to systematically apply the unintended harm typology to jointly identify potential harms and mitigation strategies with community members.

## Method

### Setting

The NCA is a UNESCO World Heritage Site located in north-west Tanzania. The Maasaiwho reside there are seminomadic pastoralists who live in close proximity to their livestock. Families live in semipermanent houses called *bomas* and move seasonally to temporary *bomas* when out grazing their livestock. The study was conducted in two wards, Endulen and Nainokanoka. The NCA is a multiple use area for wildlife, people, and their livestock, and is managed by the Ngorongoro Conservation Area Authority (NCAA). As such, there are restrictions concerning the cultivation of land, collection of firewood, digging of wells, and construction of permanent buildings.

### Data Collection

Data for this study were collected through two in-depth semistructured interviews, two group discussions, and three think tanks which were heldin May and June 2016. In-depth interviews were conducted to develop a comprehensive understanding of the water-related issues in the NCA from key informants who are deemed knowledgeable about water-related issues within the community. Group discussions were used to explore diverse perspectives and dynamics concerning water-related issues in the NCA, past and current strategies to address water quality and scarcity, and community perceptions of the potential of the BSF as a feasible and acceptable water treatment option. The interview guide covered a series of relevant topics related to water-related issues in the NCA, such as perceptions of the impact of climate change in relation to human and animal health, and the significance of water to Maasai culture. The group discussion guide probed the following issues: perceptions of water-related issues in the NCA, stakeholder engagement in water-related issues in the NCA, current strategies to address water scarcity, and current strategies to address water quality. The in-depth interviews and group discussions were conducted in English by members of the transdisciplinary research team which includes Tanzanians, Canadians, and Norwegians.

Think tanks were used to present the study and the BSF to community members to engage them in the systematic identification of concerns around introducing this technology as well as brainstorming mitigation strategies to minimize potential unintended harms. The think tank method developed by Allen-Scott and colleagues was informed by systems thinking (WHO, 2009). In this study, we used a tailored approach to suit our community-based study which included involving diverse stakeholders in collective brainstorming, discussing possible intervention effects, and using this information to adapt and redesign the intervention, as described in greater detail below.

To ensure a common understanding of the purpose and main components of the project, Step 1 involved the principal investigator presenting the objectives and methods of the BSF pilot study. This was followed by a question-and-answer session concerning the technicalities of the BSF; additionally, a picture diagram of the BSF was circulated to participants with another opportunity to clarify and describe the way the BSF worked. After this introduction, Step 2 involved a collective brainstorming session to identify potential worries and concerns about implementing the filter in the NCA. In Step 3, a discussion of the role of potential underlying factors and their interactions within the evaluation context unfolded, with the main worries concerning implementing the BSF being subsequently ranked by participants according to importance. This was followed by Step 4, which had a solutions-oriented focus whereby the group brainstormed potential mitigation strategies for each worry or concern that was identified. Finally, Step 5 culminated in an aspirational conclusion during which participants expressed their hopes for their family and community in the future.

The think tanks were conducted in a mixture of Swahili and Maasai and translated by local members of the research team to accommodate participant preferences. Interviews, group discussions and think tanks lasted between 1 and 1½ hours and took place under trees or in shaded areas, and with refreshments provided, according to local customs and preferences.

### Sampling and Recruitment

Participants were recruited with the assistance of the local project coordinator and sampling was a mix of purposive and convenience sampling. For instance, key informants were invited to participate in the study based on their role and knowledge regarding the study questions, while others were based on convenience with the aim to ensure diversity in community perspectives. The composition of the think tanks varied in terms of the occupation, gender, and position of participants in the community. An overview is provided in Table 1.

**Table 1.** Overview of Think Tank Composition.

Think Tank	Composition	Number of Participants (*n* =)
1	Diverse community perspectives, village executive officer, representative from the Pastoralist Council.	10
2	Local women’s group.	15
3	Key stakeholders (Technician from hospital, staff from a past water project, employee from the local water committee, three identified as ‘respected and influential leaders’ within the community).	11

### Data Analyses

Data analyses were iterative in the field and involved debrief sessions within the research team after each discussion. Identification of emerging themes and suggestions for potential probes in future discussions were integral to the data analysis process. All discussions were transcribed verbatim by two members of the research team to ensure rigor in the process. All data were analyzed and compared against field notes as a form of triangulation to strengthen trustworthiness. In-depth interviews and group discussions were both analyzed using thematic content analysis (Braun & Clarke, 2006; Hsieh & Shannon, 2005), facilitated by the qualitative analysis software program NVivo 11.4.0. Categories were derived from the data, not based on predetermined codes. Data from the think tanks were analyzed using directed content analysis due to an explicit focus on validating and extending the unintended harms typology. The research team included local members of the community which helped to ensure that cultural and linguistic nuances were grasped in data collection and analysis. These data formed the basis for two unpublished master’s theses (Hovden, 2017; Paasche, 2017). Findings from the study have been subsequently shared and discussed with the community members as well as study participants.

### Ethical Considerations

Ethical clearance was obtained from three ethics committees: the University of Calgary’s Conjoint Health Research Ethics Board (CHREB), The Tanzania National Institute for Medical Research (NIMR), and the Norwegian Center for Research Data. All participants were informed in advance about the purpose of the study with permission being obtained before any questions were asked. Permission was also granted to audio-record the sessions. Informed oral consent was obtained by a local member of the research team in the participants’ preferred language: Swahili, Maasai, or English. To maintain confidentiality, participants are referred to as female or male participant and by the participant’s occupation or position in the community.

## Results

Through in-depth interview and group discussion, the study first sought to develop an understanding of the NCA context, community perceptions, and salient themes related to water scarcity and water quality. Insights gleaned from these perspectives contribute to understanding the community and household context in which the BSF will be implemented. The results from the think tanks are then presented to focus on identifying the community concerns and proposed mitigation strategies for consideration in the BSF implementation.

### Understanding the Broader Context: Community Perceptions Related to Water Scarcity and Water Quality in the NCA

#### “The changes of years”—Harsher conditions now than in the past

Participants spoke at length about climatic conditions now as compared with conditions in the past, and the *Kimaa* word to describe “climate change” was identified as “*ingibelekenyat oo larin*,” which means “the changes of years” when directly translated into English. Discussion focused on conditions in the past (formulated as more than 10 years ago in the interview guide), which included more rain and green grass, greater availability of water, and less disease than the present.

According to the traditional leaders, climatic changes are of great concern to the Maasai.

They are just surprised; what is happening, what is going on? Because if they take the history, the long back history, there’s nothing like this. A long time ago, death yes, there is death, but people are healthy, they have a lot of cattle, they have a lot of rain (male participant, interview with traditional leaders).

. . . now, because there is, there is a shortage of water or no water at all and that’s why it is different. Because by that time there is no many diseases but now there is lot of diseases because of people they can just get a little bit water there, but they share with animal (male participant, interview with traditional leaders).

This scarcity of water due in the community was perceived as being a potential cause of increased levels of disease, presumably related to the necessity of sharing water sources with animals.

#### Burden on women and girls

When we talk about water issues, we talk about women issues. (Female participant, think tank with community members)

Although men are considered to be the main decision-makers both at the community and household levels, Maasai women and girls were identified as the ones responsible for most of the practical water-related activities throughout the course of the day. Water scarcity also presents a vital challenge for women during menstruation which was highlighted:

In menstruation time, they got a big problem because the water is so shortage (. . .) not all of them are using pads, sometimes they are using the small clothes so the water to clean those ones is difficult. (Local woman: In-depth interview)

A host of concerns were raised regarding the risks associated with the collection of water by women and girls, such as the physical burden of carrying heavy loads of water which may cause injury. A donkey, which was referred to as *“the woman’s car” (female participant, think tank with local women’s group)*, was mentioned as a form of assistance for transporting heavy buckets of water, but only a few in the NCA have the socioeconomic means to own a donkey. Collecting water from deep wells also posed a risk as women and girls may fall into the well, which was stated as a major concern especially for pregnant women. In terms of the fairly common incidence of sharing water sources with animals, participants reported that women and girls risk running into wild animals including elephants, buffaloes, and snakes, both on their way to and from water sources.

When you go in the morning you can find wild animals, like elephants (. . .) and buffalo. (Female participant, think tank with local women’s group)

Or they may face sexual assault en route to and from water sources:

So it may be a challenge because children, people rape those girls. Yeah, sometimes living here and around. (Local woman, in-depth interview)

In addition, women and girls who spend a substantial amount of time per day collecting water mentioned a lack of time for other family-related activities, which affected their ability to take care of and develop their family or attend school.

Another dimension related to women and water relates to the spiritual role of Maasai women during periods of water scarcity. When rainfall is lacking but anticipated, participants described specific Maasai rituals which are traditionally performed by women to bring rain. The women gather to sing and walk to the rivers or the mountains where a pregnant woman, about to give birth, will lie down on her back. This act is essentially perceived to be a prayer, and when God recognizes their need for water he will provide the necessary rain.

. . . when the women walking, singing and pray for God, they can just take one of the pregnant women who is really about to give birth and then they find that place on the plainland, and then they can just tell her to lay down there at the middle of that place, laying down on her back, just to show the God her pregnant, and then the women just singing around, just pray for God and then the God can just really listen their prayer and give rain. (Male participant, interview with traditional leaders)

#### Knowledge related to water quality

With respect to water and water quality, the general level of knowledge varied widely among participants. Most participants demonstrated a general awareness of water quality as a potential threat to health and recognized this as a widespread issue in the NCA. Diseases, such as typhoid, as well as skin and eye diseases, such as trachoma and diarrhea, were identified as diseases believed to be caused by consumption or use of untreated water for hygienic reasons. Many common sources for drinking water, especially the river, were perceived as possible sources of illness and often believed to be caused by “small and bigger bacteria in the water” (male participant, rural BSF group discussion). Boiling, cloth filtration, sedimentation, and use of WaterGuard (locally available chlorination) were identified as strategies among households; however, boiling was perceived by most participants as uncommon in the area. There are several potential reasons why the Maasai reportedly do not boil their water; these include the need for large amounts of firewood, the NCAA restriction on chopping wood, and because boiling changes the taste of the water. Formative research conducted as part of Project SHINE also indicates that boiling water is uncommon in the NCA, and cloth filtration is typically only performed when the water is turbid (Henderson et al., 2016).

Although a few participants mentioned “bacteria,” viruses and other microorganisms were not directly stated as a cause for concern by any of the participants. As discussed in greater depth in the next section, *visible* microorganisms were described as being the most reliable indicator of dirty water, implying that a perception of water with low turbidity is safe water.

#### Water with low turbidity perceived as safe water

Participants often mentioned that many people in the community have the perception of *clear* water as constituting *safe* water. This was illustrated in an interview with a staff member of the Water Committee:^1^

So, one of the perceptions from the community, people that have been using water from rivers, flowing river, from dams right, so and it has a very high turbidity, it has a lot of microbes sometimes then we can see them by naked eyes, so for them when it comes now to water quality, so safe and clean water for them (is) if it lacks, it’s not turbid . . . but also if they don’t see also like moving microbes, or moving maybe worms or something which they can see by naked eye, they see that there is nothing moving like creatures moving, that one is safe. (Employee, Water Committee)

Notably, when asked about his personal view on safe water, the same participant explained that despite being aware of the risks associated with untreated water, in practice, he still views clear water as safe water.

He understands that for him, like you mentioned earlier, the water is clear. And no movement of living organisms, that water is clean and safe. However, from the media like radio, television they have been hearing that in order for the water to be safe it must be treated. So, that kind of perceptions is coming up. However, for them, it’s hundred % that (if the) water is clear and it lacks moving living things, it should be safe. (Employee, Water Committee)

#### Shared water sources with animals and implications for health

Viewed within a One Health paradigm, which highlights the inextricable link between the broader ecosystem and animal as well as human connections (Rock et al., 2009), water is perceived by the Maasai as being synonymous with life itself.

We understand that humans are in need of so many things, however water, water is life. Water is life for the animals, water is life for the human being. So, you can have everything but if you don’t have water, then there’s no life. (Employee, Water Committee)

With respect to sharing of water sources with animals, both the quantity and quality of water was perceived as a challenge to the coexistence of human and animals. In addition, although education was perceived as urgently needed in the community, the participants, who as previously mentioned were themselves generally well aware of the link between water quality and the transmission of waterborne disease, repeatedly raised the challenge of sharing water sources with animals as a primary cause of waterborne disease.

The lack of adequate water to meet the daily requirements of humans, livestock, and wild animals was also a recurring concern. Both the human and the wildlife population in the NCA have increased substantially between the 1960s and now (Galvin et al., 2008).

. . . people nowadays they have a lot of cattle and the population of people is high also that’s why. . .and then high population with animals like elephant they share the same water that’s why nowadays there’s not enough. (Male participant, interview with traditional leader)

The conflicting interests of Maasai pastoralists, their livestock, and wildlife conservation were raised as a substantial challenge given that the NCA is a UNESCO World Heritage Site that is a major source of revenue through tourism. Human use is restricted in designated areas within the NCA to prevent negative impact on the wildlife population, and participants recounted that, as a result, the Maasai are denied access to much needed water sources. As previously mentioned in relation to perceived climatic changes, the Maasai being forced to live even closer together with their livestock may have implications for zoonotic disease transmission.

Nevertheless, as pastoralists, the Maasai are accustomed to life spent in close proximity to livestock and wild animals. Participants identified several common practices to manage water sources between humans, livestock, and wild animals. For instance, participants described the separation of the river is into sections for collecting drinking water and washing clothes. In addition, designated areas exist in which people are permitted to water their livestock. Other strategies mentioned were to place a guard at important sources to help control the various uses of the river, or a scarecrow is sometimes used to scare away wild animals. Fencing was cited as the most common strategy to keep both livestock and wild animals away from water sources, although as discussed by participants, wild animals, such as elephants and buffalos, are unlikely to be deterred by such measures, while monkeys and baboons are able to climb both trees and fences.

#### Perceived challenges associated with management of water-related issues

According to traditional leaders, the NCAA has failed to deliver on their promise to the Maasai to bring water from the protected areas to an area in which there would be no conflicting interests with wildlife conservation.

During that time the communities tried to sit with the NCAA, because of that area, then the NCAA promised them that they can just go and sit and make a budget to bring water from that area to the area where there is no wild animal where they can just allow people to stay there and get the water for their own use and their own animal, but up to today nothing is done. (Male participant, interview with traditional leaders)

In addition, participants identified several issues that can be interpreted as perceived management and leadership shortcomings related to water in the NCA. For instance, the failure of key stakeholders to work collaboratively and efficiently was a recurring issue raised as a potential reason explaining the projects’ lack success in achieving their intended goals. Participants suggested that instead of collectively focusing on one large and effective project, many small and reportedly inefficient projects are present in the community. Furthermore, executive decisions that fail to engage all stakeholders in the community, as well as the poor quality of the actual work, and the quality of the materials used, were identified as concerns related to the efficiency of water projects in the area.

Perceptions of strained relations between the main stakeholders concerning water-related issues and the wider community were also a consistent theme across discussions. In one of the think tanks, a participant pointed to power imbalances between executives managing the projects and members of the community, and that community members feeling powerless to question any decisions made. Trust was a key theme, with one participant explaining that a World Bank project^2^ was supposed to supply her village with water, but due to poor management it ended up with none.

### Community Concerns and Proposed Mitigation Strategies Related to the Implementation of the Biosand Filter–Unintended Harm Typology

Against this backdrop of community perceptions regarding water scarcity and quality, the results from the three think tanks will be presented, starting with identifying potential underlying factors of the BSF implementation, and then subsequently linking the worries to the unintended harm typology. The top ranked worries will then be presented and, finally, the mitigation strategies identified by the community to tackle harm related to the BSF will be discussed.

#### Associated underlying factors regarding the implementation of the BSF technology

Two categories of underlying factors associated with potential unintended harms of the BSF implementation emerged in this study, namely, ignoring root causes and the boomerang effect. The category that was originally labeled lack of community engagement was modified here to lack of sustainability, due to responses from the participants emphasizing a need for the BSF to be sustainable in order for the project to be considered a success. Factors associated with limited and/or poor-quality evidence was not considered relevant given that data were collected in the preimplementation phase. Nonetheless, limited and/or poor-quality evidence will be relevant in follow-up phases. Implementation in an LMIC is also not relevant in this study as the BSF is specifically developed for LMICs, and especially rural areas in which water turbidity is high.

#### Ignoring root causes

Ignoring root causes refers to the failure to consider the local context in which the intervention unfolds (e.g., resources; geographic considerations, including seasonality; hierarchies; poverty; and culture). The main underlying factor found to be associated with ignoring root causes in this study was concerns regarding poverty. This concern was particularly prominent in the women’s think tank. The costs of the BSF (approximately 120,000 Tsh or US$52) was mentioned by the research team in all think tanks. Due to a lack of money, the women in the think tank expressed concern regarding the possibility of purchasing a filter if additional filters were to be sold after the pilot project.

It will be the competition because when she saw with her eyes, everyone wants to have (BSF) in her house, in her boma. (Female participant: think tank with various community members)

Although the BSF may create competition among community members, the women in the think tank discussed contrasting views, for instance, that it may not cause an insurmountable problem for those who do not receive the BSF, as there are already existing alternative practices for water treatment.

Before you bring that filter, we just used that boiling, and the filter with the cloth, we continuing to do so. So it would not be a problem for them, if they don’t have. (Participant: think tank with local women’s group)

However, upon further reflection, having the BSF was expressed as important by the same participants due to being beneficial for human health.

It will be the difference, because when they have the filter, they decrease the diseases like diarrhea and the vomiting, so it can change the life of itself. (Participant: think tank with local women’s group)

In the think tank with various community members and stakeholders, participants expressed the possibility of selling cattle as a means to buying the BSF. One of the participants in the think tank with the stakeholders provided the example that many now live in modern houses rather than traditional *bomas*, have mobile phones, and use solar chargers, which illustrated the shift of the Maasai toward a more modern community. The participant explained that when people learn about new technologies or ways to improve health, it generates interest among others as well as creates a desire and consequent demand for the new technology or strategy.

Underlying factors, such as poverty and the large distances between communities within the NCA (which pose a challenge in terms of spreading the word widely about the BSF), is associated with ignoring root causes and can lead to inequality, which may subsequently result in psychological harm. This concern was highlighted in the think tank with various community members.

Resources and the raw materials needed to construct the BSF are scarce in the NCA, and permission to bring supplies into the conservation area was identified as a concern in the think tank with various community members and stakeholders. In addition, once permission is obtained, the next challenge relates to gaining access to materials essential for the construction of the BSF. This is both due to regulations in the NCA, and local policy dynamics among community members. This is similarly associated with ignoring root causes. The project did not experience any challenges with bringing materials into the NCA; however, it was suggested that as the project expands this may become more of an issue.

Think tank participants reiterated that it is essential to be aware of the different levels of leadership within the community and the hierarchy, both from a grassroots and a stakeholder’s perspective for the project to be sustainable. Failing to engage different levels of leadership may ultimately result in unintended harms associated with ignoring root causes. Based on consultation with local leaders and stakeholders, it was decided that household selection to receive a BSF would be conducted at an open community meeting facilitated at the Village Executive Officer (VEO) headquarters, in line with community norms concerning similar projects. In the think tank with the various community members, one of the male participants stated that having the VEO take a leadership role in selecting *bomas* to receive a BSF is a culturally appropriate way of working within the hierarchy and involving the community. The participants appeared to find this decision satisfactory. However, on several occasions during the think tank, participants emphasized that although they respect the political and cultural structure in the community, they still feel skepticism toward the executives. According to the participants, community members take the lead in deciding who receives the BSF, as private individuals cannot question authorities if the project fails. This tension and exchange highlights the strained relationship between community members and project managers or executives, which is an important root cause that needs to be addressed to avoid harm.

The fact that Maasai are seminomadic pastoralists was identified as a concern in the think tank with the stakeholders and is associated with ignoring root causes. A fully installed BSF weighs around 65 kg, which can be difficult to transport and, if damaged, can potentially result in unintended harms if it not properly repaired, or if insufficient care is paid to maintaining the biological layer, which is essential for the filter to effectively function. Nevertheless, this was not raised as a concern in the think tank with various community members, the rationale being that women stay behind with the children and can maintain the filter while the men are grazing the cattle. It was also mentioned by the women that educated people do not usually shift *bomas*.

Only cattle, goats and men are the one that move. But women and children remain behind. Educated people they are not moving. (Male participant: think tank with various community members)

However, seasonality may affect the use of the BSF in other ways; for instance, when men leave with the cattle, they will not have the possibility to drink clean water if the BSF is left behind with the women. In addition, seasonality may result in water scarcity, which poses a challenge in terms of ensuring the filter receives the amount of water needed to keep the biological layer alive. This could potentially result in women and children also not being able to use the BSF.

As highlighted above, a host of considerations must be taken into account with respect to ignoring root causes. Unique contextual factors and the Maasai way of life may potentially lead to unintended consequences, which are associated with several factors stemming from Allen-Scott et al.’s (2014) typology, including harm by omission and political harm.

#### Boomerang effect

Within the context of public health campaigns, the boomerang effect often relates to warning messages (e.g., NO DIVING) and information-based interventions which can induce the opposite rather than intended attitudes or behavior (Ringold, 2002). According to Wogalter et al. (1999), cited in Ringold (2002), potential factors that may lead to an opposite effect than intended have thus far received limited attention in the literature.

A lack of adequate education, training, and maintenance of the BSF was identified as a concern in the think tank with the diverse community members and the stakeholders. The worry that people need adequate training and in-depth knowledge was highlighted; without it, participants were concerned about potential misuse of the filter as well as an increased risk of recontamination of the water. Another expressed worry related to this issue was people profiting from selling poorly constructed BSFs.

#### Lack of sustainability

Sustainability is an ethical imperative that needs to be considered and planned for at all phases of a health promotion intervention. Key questions, such as “What happens when the funding for the research ends?” and “Who will be there to support the community?” raise important issues that need to be addressed. This was particularly salient in the think tank with the various community members, with one participant remarking,

Because most of the projects is just passed away projects. They just come back like helicopter projects. They just land here today, do the next project for a short period of time, and then they disappear. (Female participant: think tank with various community members)

Without adequate leadership, participants expressed worry that some people would not be reached by the project and that the project would only benefit those living close to the village center. In the think tank with the stakeholders, the importance of having appropriate leaders in place at a policy level was highlighted as necessary for sustainability and growth of the project.

#### Mitigation strategies

Several possible mitigation strategies emerged from the think tanks which were discussed and subsequently ranked to correspond to the top ranked worries related to the BSF implementation.

##### Worry 1: Inequality stemming from who receives a BSF

The imperative of grassroots leadership and minimal involvement from executives was repeatedly emphasized. This was a consequence of the skepticism toward the executives holding all the decision-making power regarding distribution of the BSF, which participants believed may lead to inequality. Concerns were expressed that executives may fail to prioritize households who need the BSF the most yet lack the means to pay for one. Nevertheless, one solution put forth to mitigate this potential inequality was to implement safeguards to ensure transparency and cross-checking to see whether there is a fair and equitable distribution of BSFs in the community.

A strategy proposed by the participants of the women’s think tank was the possibility to share clean water with neighboring *bomas* if someone had the money to purchase one.

I have to pay maybe for her or to help her, and then, have in my house, I collect other people, I give them water through the filter. (Participant: think tank with local women’s group)

##### Worry 2: Lack of/or poor local leadership and support of the BSF project

This is partly related to the first worry, as it centers around the importance of establishing trusted leadership at the grassroots level. For the participants, the importance of ownership and local leadership with respect tothe BSF project was expressed as essential for the BSF project to be sustainable.

Several strategies were proposed as a means to mitigate concerns that a lack of local leadership and support may lead to barriers to expanding the project, for instance, regarding transport of materials into the NCA, such as the tank. The need to have multiple stakeholders in place advocating at different levels within the structure was stated as key for sustainability, which in turn will affect the availability of materials. The group suggested having a spokesperson (health promoter/health officer) for the BSF who can speak on behalf of the community.

If health promoters, somebody that is like health officer in Endulen involve about this things, then it will be easier to use him for the process of the government, if there is any possibility of the government to support this project. (Technician working at the hospital: think tank with various stakeholders)

According to a traditional leader, having key leaders at the local level in both the Endulen and Nainokanoka area, who can promote and advocate the BSF to policy leaders in the NCAA, was put forth as a strategy that can potentially mitigate the issue of getting the raw materials needed for constructing the filters into the NCA. He advised that a written letter from the village officer should be given to the Pastoralist Council (PC) to present the project at a NCAA meeting. With further funding, access to materials would potentially increase, giving more people in the NCA the opportunity to access a filter.

During the think tank discussion, a concern experienced by the research team regarding the procurement of sand required for the BSF was shared with the participants, in order to give them a concrete example of some of the challenges regarding accessing materials for the filter. The team experienced political challenges, as they were unable to obtain an adequate supply of sand from areas where the BSF project was being implemented and had to approach other communities. This posed an issue that required negotiation, and if the project were to scale up, might face similar challenges.

Potential mitigation strategies to address this concern included providing information to nearby communities about the BSF evaluation, reassuring them that the BSF is for all people living in the NCA and that areas that contribute by providing sand are prioritized in future BSF installations.

Overall, key leaders and politicians in the think tank advised that there are several important bodies within the community structure with which to engage to ensure the project’s accountability and sustainability, such as the PC, the NCAA, and the Development Office. This may also result in additional funding and support for other communities if the pilot project is successful.

##### Worry 3: Lack of adequate education and training to use the BSF

Strategies to mitigate concerns regarding low levels of education within the community emphasized the importance of a proper implementation phase using adequate education and training of health promoters and BSF technicians.

The education piece, more people will be educated and raise awareness so that people will understand the BSF and the importance of having safe and clean water. And it will be more successful in the second hand if there is adequate implementation. (Male participant: think tank with various community members)

Furthermore, participants identified education as key for long-term sustainability, and expressed that if they would be given a BSF, they could share knowledge of ways to use and maintain the BSF within the community.

You have to give them, to teach them, and to give them and how to using them to spread word to other people. So they need to use, and to know, and they can spread for other people. (Participant: think tank with local women’s group)

Involving school children and teachers was also proposed as a strategy to increase knowledge of the BSF, which would also enhance the sustainability of the project. Furthermore, to secure proper leadership, learning from the successes and shortcomings of previous projects, such as the World Bank project, was identified as a way to mitigate this concern.

## Discussion

This study addresses two crucial gaps in the literature; first, by contributing to understanding water-related issues, such as scarcity and quality from the perspective of pastoralists. Second, the study provides novel methodological insights into the ways that meaningful and sustained dialogue through a think tank method with the community can promote the effective introduction of a low-cost, low-tech household water treatment option, such as the biosand water filter, with an explicit focus on identifying and mitigating potential unintended harms. The think tank approach as a unique method to secure authentic community engagement at all phases in the research process also contributes new insights into approaches to reflexively involve the local community in the development and implementation of a public health intervention, which is an important issue which has thus far received limited attention (Johnson & Schoonenboom, 2016; Wigginton et al., 2020).

### Community Perceptions Related to Water Scarcity and Quality

With respect to water scarcity, the most prominent concerns that emerged relate to perceived climate change, the burden on women and girls, insufficient amounts of water to meet the needs of both humans and animals, as well as shortcomings in terms of management and leadership of water-related issues. The change in climate in the NCA, resulting in less rainfall and increased water scarcity as well as the sharing of water sources with animals, may result in increased levels of waterborne disease in the community. As zoonotic disease transmission increases when humans and animals are forced closer together depending on the same water sources (Mazet et al., 2009), a scenario of increased water scarcity is indeed likely to cause increased levels of waterborne disease. It is difficult to draw conclusions about trends in climate changes, such as annual rainfall on the African continent, due to insufficient observational data (Niang et al., 2014). Nevertheless, data from the past century indicates “very likely increases over parts of eastern and southern Africa” (Niang et al., 2014, p. 1209). At the same time, Lyon and DeWitt (2012, cited in Niang et al., 2014) identified a decrease in rainfall in the March–May season in eastern Africa, which is consistent with the perceptions of participants concerning water scarcity and the length of the rainy season in the NCA. Whether concerns regarding water scarcity are perceived or actual is an issue that merits further study.

However, it is vital to consider the community concerns around decreased amounts of rainfall and available drinking water leading to waterborne disease, as they may have implications on the perceived need for water treatment options, such as the BSF. This is highlighted by the findings from a study conducted in India, which found that the perceived need for water treatment is one of several factors affecting the willingness to pay resulting in demand for the BSF (Ngai & Fenner, 2014). In light of the perceptions of less rain, less available drinking water, and the current increase in waterborne disease in the community in comparison to the past, water treatment options, such as the BSF, are more likely to be perceived as beneficial and desirable.

Closely related to the issue of water scarcity, concerns were raised regarding the physical burden and associated risks related to the collection of water which impact women and girls in particular. This concern is consistent with findings from other studies which indicate that women and children in Tanzania spend between 2 and as much as 7 hours every day collecting water, with a heavier burden in remote areas (WaterAid, n.d.). Carrying heavy loads of water takes a toll on the body, for instance, in terms of caloric expenditure, injuries to the back, neck or joints, risks of falling, and assault and attacks (i.e., rape or attacks by wild animals) (Sorenson et al., 2011). In addition, regarding girls and young women, studies indicate that time spent assisting with water-related activities at the household is time that may otherwise have been spent in school (Sorenson et al., 2011). In Tanzania and other countries, improved access to water has been shown to increase school attendance by up to 15% (United Nations Children’s Fund, 2006).

Concerning the issue of water scarcity and the impact on women and girls, adoption of the BSF in households is not likely to reduce the extra burden, given that household water treatment options do not increase access to water, and water availability will continue to pose an issue whether or not a household has a BSF to treat their water. Nevertheless, compared with the present situation in which the BSF is integrated as a part of the women’s everyday life, having a BSF may reduce the time spent collecting firewood to boil water, thereby giving space for engaging in other productive activities related to work and school (Kaiser et al., 2002).

The final concerns that emerged related to water scarcity were challenges related to the managementof water-related issues in the NCA. Current efforts to increase access to water were perceived as inefficient due to poor coordination among the main actors and other stakeholders. In addition, a strained relationship between the executives and the community was identified, in terms of an apparent lack of trust. These fundamental issues may have important implications affecting the potential for BSF adoption and expansion within the NCA.

The main concerns with respect to water quality that emerged were related to overall knowledge levels in the community. Although participants demonstrated a general level of awareness of water quality and potential transmission of waterborne disease, concerns about the overall knowledge levels in the community at large were repeatedly raised, with the most common perception of safe water being incorrectly characterized as water of low turbidity.

This finding is also relevant to consider in relation to identified health concerns related to the necessity of sharing water sources with livestock and wild animals. Viewed from a One Health perspective, the relationship between the Maasai, their livestock, and wild animals may have deeper implications. The argument is simply that despite Maasai not expressing their knowledge using words, such as “bacteria,” ‘virus “pathogens,” “contamination,” or “water-borne disease,” the average Maasai does have a reasonably high level of awareness of contaminated drinking water as a potential cause of disease. This is evidenced by local efforts to manage human and animal use of water sources. Based on this, when considering issues such as the acceptability and feasibility of the BSF, it becomes highly important to situate health-related issues, such as the transmission of waterborne disease in the NCA, within a One Health perspective, which takes into account the importance of Maasai traditions, wisdom, and knowledge.

### Unintended Harms Associated With the BSF Intervention

The findings of this study illustrate that the complexities of evidence, context, potential boomerang effects, and community engagement are important considerations when it concerns mitigating physical, psychosocial, economic, and cultural unintended harms. Using the unintended harm typology as a conceptual framework helped contribute to a broader understanding of identifying and mitigating potential negative consequences and harms. Implementing the BSF in a unique context, such as the NCA, requires systematic consideration of both context-specific unintended harms and underlying contextual factors. In addition to interviews and group discussions with key members of the Maasai community, by specifically facilitating think tanks, meaningful and engaged dialogue about potential harms and mitigation strategies associated with the implementation of a new technology contributed to the overall ownership and sustainability of the project. As a method, the think tank approach can serve as an important intervention planning and evaluation tool that fosters community involvement at all phases to ensure minimal harm to participants, and also enhances local ownership and sustainability.

In this study, the most salient community concerns associated with potential unintended harms of the BSF were inequality and a lack of sustainability of the project due to poor leadership and education. These concerns relate to the large geographic area of the NCA and people being geographically spread out across rural and periurban areas. This poses a challenge in terms of reaching as many inhabitants as possible, particularly the most vulnerable who would benefit from the BSF the most. Ignoring root causes, such as the large geographical areas within the NCA and poverty, may lead to harm by omission and psychosocial harm. For instance, the people who stand to benefit the most from an intervention are often least likely to receive them (Lorenc et al., 2012). Even if the pilot intervention demonstrates promising results, including improved health and well-being within the population, the intervention may still have generated inequality. Therefore, focusing on identifying unintended harms while concomitantly developing mitigation strategies in a consultative and systematic way is vital to ensure the intervention prevents exacerbating existing inequalities or creating new ones in the community.

Mitigation strategies to tackle inequality should be centered on positioning community members as responsible for determining who receives the BSF in partnership with community leaders. This circles back to the importance of a contextual understanding and working within the hierarchy of the local setting when introducing a new technology. It also points to the need to engage the local population at the grassroots level as it is they who have the skill set and position required to bring the project forward as well as ensure equity and sustainability.

In the future, mapping households to identify those that are in real need for a BSF can be a way to mitigate inequality in collaboration with a grassroots leadership. Involving community members in efforts to promote and advocate for the BSF contribute to the overall sustainability and scale up of the BSF project. In this way, it is possible to mitigate both harm by omission and psychosocial harm inflicted by the BSF evaluation.

As mentioned, a lack of trusted leadership at the grassroots level was a prominent concern in the think tank with various community members. Any effort to expand the BSF without concomitant political support or involvement in the project, both at the grassroots level and policy level, may ultimately result in political harm. The importance of Project SHINE’s collaboration with policy leaders was stated as crucial for the project to be sustainable. If bodies, such as the Pastoralist Council, Community Development Office, and the NCAA, are engaged in the project, this can potentially mitigate political harms associated with future BSF scale-up. Engaging with policy stakeholders is important to secure additional funding to expand the BSF as well as to increase the accessibility of materials both within the local context and outside of the NCA. Leadership at a grassroots level is important for the wider community to be involved ensuring equal distribution of the BSF. Trusted leadership is important at both levels and may contribute to a mitigation of political harm and harm by omission.

Insufficient training on maintenance of the BSF was a prominent concern expressed by the participants. In a study on household water treatment and safe storage practices, it was found that training was essential for ensuring correct use of household water treatment systems, regardless of the degree of user-friendliness of the technology (Ojomo et al., 2015). Inadequate education and training may lead to a potential boomerang effect, which is associated with both physical and psychosocial harm. Although the BSF is considered to be low-tech, the complexity of the components may still be challenging to understand for some. Due to the complexity related to the mechanism of the biological layer, sufficient knowledge is required to build, operate, and maintain the BSF. A study which investigated the long-term effects of BSF distribution in Ethiopia found that low usage rates and poor performance were associated with quality of maintenance, lack of education, training, and support (Earwaker & Webster, 2009). This may result in reduced effectiveness and poorer water quality or, in the worst case, a breakthrough of pathogens leading to illness. Ongoing education and the application of a train-the-trainer model within the project was also identified as an important strategy to increase ownership and sustainability within the project.

Psychosocial harm (not mentioned in the think tanks) may occur among those who also receive education in regard to the BSF as this increased awareness may lead to feeling helpless or disempowered, especially if one lacks money to buy one. Psychosocial harm was clearly identified in Allen-Scott’s et al.’s (forthcoming) study on the harmful unintended effects on Project SHINE’s implementation of locally and sustainable strategies to improve sanitation and hygiene. Together with secondary school students and teachers, psychosocial harm was identified as a result of frustration and perceived disempowerment, based on the inability to implement knowledge due to resource barriers and power dynamics within the community. These identified underlying factors might be found in the present study in which participants identified similar worries. For instance, power dynamics and the concern expressed in the present study regarding the importance of having appropriate leadership in place to promote the project further. By providing quality training together with proper leadership and management, this can contribute to mitigating the boomerang effect, which in turn can reduce, among other factors, physical and psychosocial harm as a result of the BSF implementation.

A lack of money (poverty) was a prominent concern that is associated with economic harm as well as psychosocial harm inflicted by the BSF evaluation. Despite the BSF being considered a low-cost technology, many Maasai still cannot afford one. Therefore, a model which ensures that those most in need receive subsidies or other forms of assistance to access a filter is important to mitigating disparities in access. On the other hand, the price of purchasing a BSF may potentially lead to other harms, such as economic, psychosocial, and physical harms.In addition, increased modernization of the community particularly closer to the village center may result in even greater inequality among those with low socioeconomic status, due to limited access to technologies, such as mobile phones, solar power, and the BSF.

The scope of the project was stated as a concern by the participants, although the research team strived to clarify that the project was a pilot study intended to determine the feasibility and acceptability of the filter. However, the misconception may be an issue due to past experiences within the community with “helicopter projects” (Smith, 1999) that drop in for a limited time only and lack community engagement and sustainability. Distrust within the community toward key stakeholders, both within the project and in the community, and the leadership itself must be carefully addressed.

Resource barriers were a prominent concern among participants. A limited availability of resources to construct the filter may lead to psychosocial harm, due to powerlessness. A lack of resources will also affect sustainability; it is challenging with the scope of a pilot project to tackle underlying factors (such as a lack of money and resources), potentially leading to economic, psychosocial, and physical harm. Nevertheless, one mitigation strategy expressed by the women’s group was the ability to share the BSF among households. According to Earwaker and Webster (2009) who conducted an evaluation of the long-term sustainability of BSF in rural Ethiopia, it was not only the households owning the BSF that benefited from the filter. Friends, neighbors, and workers also used the water produced from the BSF. As mentioned, to increase the availability to resources, the importance of collaboration with policy leaders was a prominent mitigation strategy.

This study highlights a need for further research on unintended harms; both qualitative and quantitative approaches are needed to follow up on issues associated with unintended harms and mitigation strategies associated with BSF implementation in other areas with similar water concerns. To that end, we note that a follow-up substudy has been conducted by the team to assess perceived long-term community acceptability and feasibility (Paasche et al., forthcoming).

## Conclusion

It is essential that public health interventions are developed in partnerships with communities and tailored to the unique social, cultural, economic, and political context to be effective. It is also vital that interventions engage communities in identifying and mitigating potential unintended harms of well-intentioned interventions. The unintended harms typology has provided this study with a useful framework for facilitating careful and systematic consideration of the underlying factors associated with the implementation of a new technology to improve health outcomes in a vulnerable population. Future studies with a similar focus may benefit from the inclusion of an unintended harms lens as well as the application of the think tank method as an explicit strategy to authentically engage the community in the development, implementation, and evaluation of a proposed intervention.

## Notes

1. The Water Committee was described as a local consultative body related to water-related projects in the Endulen community.

2. We were unable to identify any information online about this past World Bank water project.
